# Special Issue “Design and Application of Additive Manufacturing”

**DOI:** 10.3390/ma15134554

**Published:** 2022-06-28

**Authors:** Rubén Paz

**Affiliations:** Mechanical Engineering Department, Campus de Tafira Baja, Universidad de Las Palmas de Gran Canaria, 35017 Las Palmas, Spain; ruben.paz@ulpgc.es

Additive manufacturing (AM) is continuously improving and offering new opportunities in the manufacturing industry. The advantages of AM, such as design freedom, reduction in material waste or low-cost prototyping, can be exploited to improve or replace traditional manufacturing methods. To realize this, the combination of design, materials and technology must be thoroughly analyzed for every specific application. This Special Issue presents several studies related to the design and application of AM technologies, covering different technologies, methods and applications useful for the promotion and uptake of AM technologies.

Nam and Pei [1] analyzed the influence of some process parameters (print pattern and infill density) of material extrusion AM (MEX) in the recovery properties of polylactic acid (PLA) samples (4D printing). The ‘Quarter-cubic’ pattern with a 100% infill density showed the best recovery result, whereas the ‘Cubic-subdivision’ pattern with a 20% infill density demonstrated the shortest recovery time. On the other hand, a high recovery temperature and high infill density resulted in better recovery, and a low temperature and low infill density resulted in poor recovery.

In the research by Paraschiv et al. [2], laser defocusing in the selective laser melting (SLM) process (powder bed fusion, PBF) was investigated to assess the influence on the surface quality, melt pool shape, tensile properties, and densification of the produced samples (IN 625 material). It was observed that the melting height increased while the melting depth decreased from positive to negative defocusing. The use of negative defocusing distances yielded the maximum density and ultimate tensile strength.

In [3], a new method for the automatic detection of powder spreading defects in the SLM process was developed, with a high recognition rate and rapid detection speed, which could not only meet the SLM forming efficiency, but also improve the quality of the formed parts through feedback control.

Kusano et al. [4] proposed a novel calibration strategy for the heat source of a finite element thermal simulation model of the SLM process, thus improving the accuracy of the temperature field estimated during the process. The new calibration strategy combined with the finite element thermal simulation model may be useful to understand the complex thermal history applied in the layer of metal powder (which cyclically melts and solidifies) and that affects the microstructure, material properties, and performance.

Regarding the finite element analysis of the mechanical properties of porous structures (scaffolds made by AM for tissue engineering), Vega et al. [5] developed a new geometry-based modelling methodology to model the deposited part in MEX AM (starting from the manufacturing file), which enabled the mechanical simulation of the deposited geometry by finite element analysis. They compared this methodology with a voxel-based modelling technique in terms of the accuracy of the simulations (with respect to experimental mechanical results) and computational efficiency. Geometry-based modelling performed better for simple or larger parts, whereas voxel-based modelling was more advantageous for small and complex geometries. Both methodologies led to certain inaccuracy in the prediction of the mechanical properties, but enabled the comparison of different deposited geometries, which is useful for the design optimization of porous structures.

Adiaconitei et al. [6] manufactured a closed impeller for high-pressure mechanically pumped fluid loop (MPFL) systems by SLM, fulfilling the quality requirements and avoiding the technical challenges related to the conventional manufacturing process, thus demonstrating the feasibility of AM for this space application.

Zhong et al. [7] investigated the deformation mechanism and energy absorption behavior of 316 L triply periodic minimal surface (TPMS) structures with uniform and graded wall thicknesses fabricated by SLM. They observed that the stress level and length of each plateau in the deformation process could be adjusted by changing the wall thickness and position of the barrier layer between different segments. The gradient design of TPMS structures may find applications where the energy absorption requires a double-level feature or a warning function.

In [8], a flexible pressure sensor (widely used in human motion, robot monitoring, and medical treatment) was developed with a flat top plate and a microstructured bottom plate, both made of polydimethylsiloxane (PDMS) molded from a 3D-printed template. The contact surfaces of the top and bottom plates were coated with a mixture of poly (3,4-ethylenedioxythiophene) poly (styrene sulfonate) (PEDOT:PSS) and polyurethane dispersion (PUD) as stretchable film electrodes with carbon nanotubes on the electrode surface. Using digital light processing (DLP), the fabrication of the sensor was rapid and low-cost.

In [9], the authors optimized a hip implant that reduced the strain shielding effect, using the solid isotropic material with penalization (SIMP) topology optimization method. Finite element analyses and experimental measurements (hip manufactured with 316L-0407 stainless steel in PBF technology) were conducted to measure stem stiffness and predict the reduction in stress shielding. The developed topology optimization process enabled compliant hip implant design for more natural load transfer, reduced strain shielding, and improved implant survivorship.

Lam et al. [10] examined SLM-fabricated 15-5 PH steel with the 8% transient–austenite phase towards the fully reversed strain-controlled low-cycle fatigue (LCF) test. The cyclic deformation response and microstructural evolution were investigated.

In [11], ABS, PETG, and PLA polymers, which are common in MEX, were joined to grit-blasted aluminum substrates. They demonstrated the suitability of a polymer and a thermal processing condition to form a polymer–aluminum joint through MEX.

Finally, in [12], the authors applied topological optimization and a technological co-design to combine the advantages of 3D model printing (PMMA material by Binder Jetting technology) and conventional investment casting production methods. The PMMA material was more accurate than standard injected wax models, but the surface quality of the printed model was significantly lower.

## Data Availability

Data sharing not applicable.
